# Novel AI-Assisted Quest-Based Therapeutic Framework for Enhancing Engagement and Executive Functioning in Attention-Deficit/Hyperactivity Disorder (ADHD): A Case Report

**DOI:** 10.7759/cureus.111426

**Published:** 2026-06-24

**Authors:** Brody Montoya, Christian F Mauro

**Affiliations:** 1 Psychiatry and Behavioral Sciences, Duke University, Durham, USA

**Keywords:** artificial intelligence (ai), attention-deficit/hyperactivity disorder (adhd), child and adolescent mental health, gamification in healthcare, individual therapy

## Abstract

Youth with attention-deficit/hyperactivity disorder (ADHD) may experience persistent barriers to engagement in individual psychotherapy despite adequate pharmacologic treatment and evidence-based therapeutic content. Existing gamified approaches often rely on fixed protocols, specialized materials, or group-based formats to address interfering symptoms such as distractibility, low frustration tolerance, and difficulty sustaining mental effort, with mixed feasibility and clinical outcomes. This case describes a novel, individualized gamified intervention designed to address limitations of existing approaches and support ADHD treatment engagement.

An otherwise healthy 11-year-old male with ADHD, combined type, was referred for outpatient psychotherapy to address difficulties with organization and emotional self-regulation while receiving methylphenidate extended-release 18 mg daily. Early sessions used established approaches, including gamified interventions, but were marked by distractibility, fidgeting, and disengagement. Treatment was reorganized into a structured, therapist-led, quest-based gamified framework delivered across 21 completed 60-minute sessions over approximately 24 weeks. ChatGPT-4o was used to assist with narrative creation and maintenance and to support integration of therapist-selected psycho-education, behavioral skills training, cognitive-behavioral strategies, mindfulness, and parent management principles into a tabletop role-playing structure. Over time, the patient demonstrated progressively improved engagement, emotional awareness, organization, and coping skills, corroborated by parent-informed improvements on the “Hyperactive/Impulsive” and “Conduct” items of the National Institute for Children’s Health Quality (NICHQ) Vanderbilt Assessment Scale, third edition.

This case illustrates how therapist-led gamification may function as a pragmatic delivery framework for evidence-based individual psychotherapy in youth with ADHD when engagement is a primary treatment barrier. The intervention emphasized therapist-guided skills coaching, flexibility, narrative reinforcement, and repeated practice. A large language model was used to make the intervention feasible, primarily as a preparatory narrative support tool under full therapist oversight. Further study is needed to evaluate the feasibility, acceptability, and generalizability of individualized gamified psychotherapy frameworks in outpatient ADHD treatment.

## Introduction

As a co-treatment with medication, psychotherapy for attention-deficit/hyperactivity disorder (ADHD) is supported by robust evidence to improve functional outcomes in youth [1]. Counterintuitively, individual therapy for ADHD requires sustained attention, intrinsic motivation, and tolerance for structured, verbally mediated sessions. There is good evidence for skills-based approaches, such as Organizational Skills Training and Computer-Assisted Cognitive Behavioral Therapy, for individual therapy. However, especially in children and pre-adolescents, most evidence is for psychosocial interventions in ADHD, such as parent management training [2-4]. Individual preferences, pharmacotherapeutic tolerance, and parental availability leave a subset of medicated youth with ADHD with some symptomatic control, but with remaining struggles to engage sufficiently in available psychotherapeutic formats due to core features of ADHD [5]. When poor engagement becomes a primary barrier to treatment delivery, how can clinicians adapt the structure and delivery of psychotherapy without abandoning individualized approaches, therapeutic intent, or clinical rigor? This clinical perspective examines engagement difficulties as a common bottleneck in psychotherapy in the case of a pre-adolescent male with ADHD, combined type, and explores how structured, therapist-led, quest-based gamification may function as a pragmatic delivery framework to support psycho-education, mindfulness, and skills acquisition when conventional approaches are difficult to implement.

## Case presentation

An otherwise healthy 11-year-old male with a recent diagnosis of ADHD, combined type, was referred for outpatient psychotherapy to address persistent difficulties with organization and emotional self-regulation [6]. He was treated with methylphenidate extended-release 18 mg once daily for 12 weeks by the time of referral. This dose led to notable improvements in school-based symptoms, including improved grades and school behavioral reports, though he experienced a mild decrease in appetite. He remained on the same medication dose throughout therapy.

Initial interviews clinically corroborated the existing ADHD diagnosis. During initial diagnostic sessions, the patient was notably distractible, fidgety, and minimally engaged, expressing limited interest in therapy. Brief incorporation of simple, developmentally appropriate, tabletop games during interviews temporarily improved participation. However, early attempts to deliver skills through worksheets, structured activities, and existing therapeutic games were followed by renewed disengagement. Conversely, the use of non-therapeutic games captured the patient’s attention but proved overly distracting, interfering with the acquisition of therapeutic concepts.

**Figure 1:**
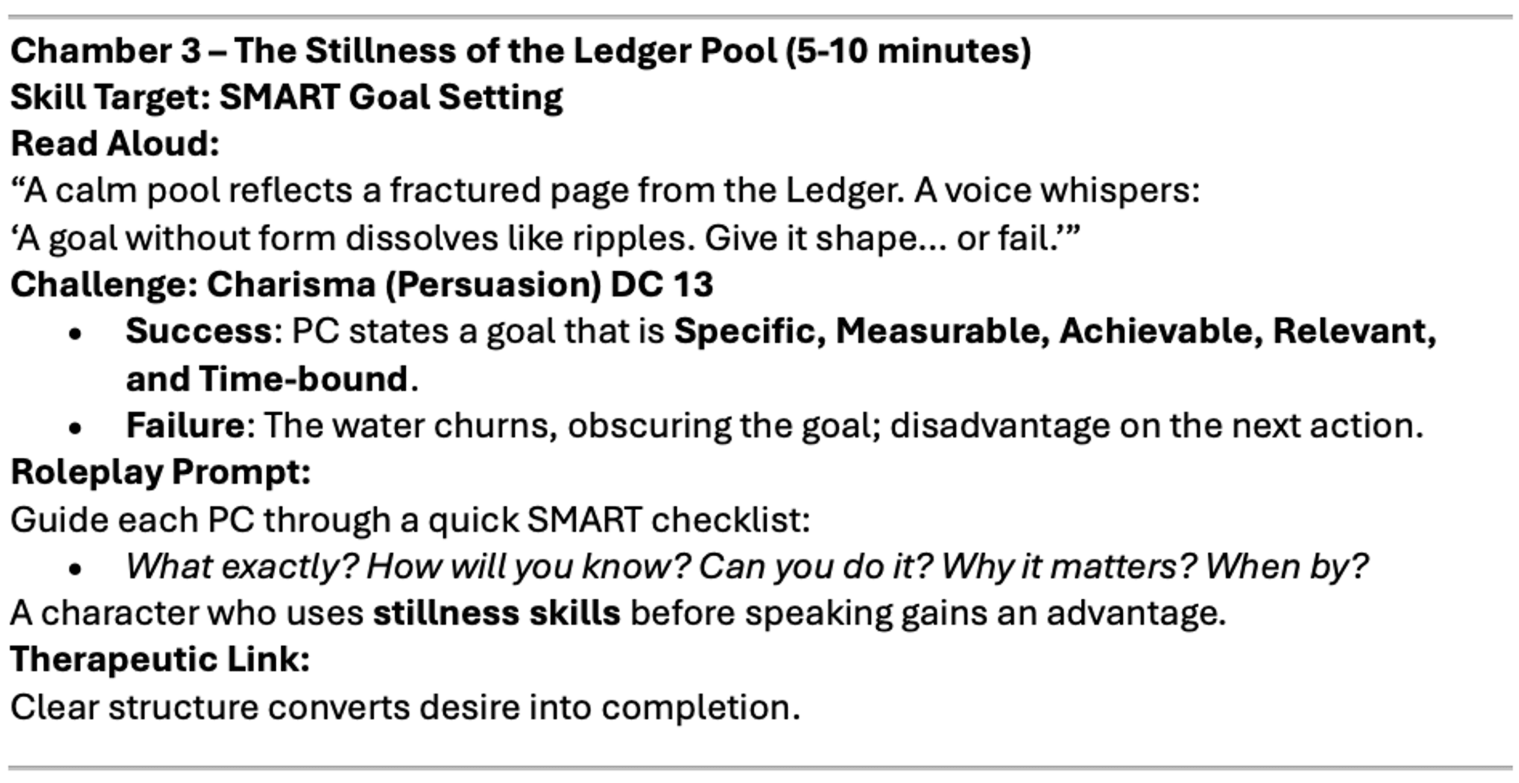
Quest Narrative and Outcomes Determination Sheet for Task Two (“Making a SMART Goal”) in Quest Four (“A Paper Storm in Tumuldur’s Library”) Example of therapeutic encounter from Quest Four (“A Paper Storm in Tumuldur’s Library”), the fourth therapist-guided narrative module within the intervention framework. In this exercise, the patient’s player character (PC) engages in a SMART goal-setting activity embedded within a fantasy roleplay scenario (“The Stillness of the Ledger Pool”). “DC” refers to “Difficulty Class,” a tabletop role-playing game mechanic representing the threshold for successful completion of a challenge. “Charisma (Persuasion)” refers to a narrative communication-based skill challenge adapted from tabletop gaming systems. Figure formatting and narrative structuring were developed using ChatGPT-4o (OpenAI, San Francisco, CA, USA) and subsequently reviewed and modified by the authors.

Given this pattern, therapy was reorganized around a structured, therapist-led, gamified framework designed to integrate targeted concepts and skills within a narrative, task-based format (Figure 1). The treatment spanned approximately 24 weeks, consisting of 21 completed 60-minute sessions, including diagnostic assessments, game setup, conceptual and skills acquisition sessions, parent training, reviews, and termination. Adaptations were made to address missed sessions and emerging behavioral concerns, with planned skills folded into subsequent sessions as needed. Gameplay materials were minimal and feasible for both in-person and tele-health visits.

Over the course of treatment, the patient appeared more consistently engaged during sessions, with fewer observable disruptions, and gradually improved competency when practicing emotional awareness, organization, and coping strategies. At the final session, parents reported 1-2-point relative improvements on several “Hyperactive/Impulsive” and “Conduct” items of the National Institute for Children’s Health Quality (NICHQ) Vanderbilt Assessment Scale, third edition, using authorized, licensed report forms [7,8]. They also report improvements in behavior and emotional regulation at home, including reduced sibling conflict, and moderate improvements in physical organization. At termination, the patient described therapy as “fun,” endorsed greater perceived control over anger, and expressed openness to returning to therapy in the future if difficulties re-emerged.

## Discussion

The goals of the intervention were to reorganize therapy into a structured, cohesive, therapist-led, gamified framework that serves as a delivery mechanism for evidence-based content. The intervention was required to meet several practical criteria: it needed to be inexpensive; feasible for both in-person and telehealth delivery; flexible enough to accommodate missed sessions or emerging concerns; and sufficiently engaging to maintain attention while still allowing for repeated skills practice and mastery. Some existing gamified approaches to ADHD psychotherapy rely on fixed protocols, specialized materials, or fully virtual formats, which may limit flexibility or therapist involvement in certain clinical settings [9-11]. Prior reports describing the use of tabletop role-playing games in therapy have most often focused on group settings or internalizing symptoms, leaving less guidance for individual outpatient ADHD psychotherapy [12]. This intervention drew on elements of prior therapeutic tabletop role-playing games and adapted them to create an individualized, developmentally appropriate structure for evidence-based outpatient ADHD psychotherapy [13-14].

The game targeted two domains commonly impaired in youth with ADHD: organization and self-regulation. Consistent with established treatment recommendations, the intervention incorporated psychoeducation, behavioral skills training, cognitive-behavioral strategies, and parent management principles. Targeted concepts and skills included emotion identification, utilization of supports, physical organization, task prioritization and breakdown, goal setting, mindfulness, cognitive restructuring, physical coping strategies, and reinforcement systems [2-5].

The game was organized around an overarching narrative in which a playable character progressed through a series of quests within a fictional world. Early in treatment, the patient identified personal strengths and challenges and distributed these across multiple characters, allowing different roles (e.g., leader, supporter) to be explored over time. Skills acquired during therapy were incorporated into the characters’ abilities, providing a concrete representation of progress. The therapist functioned as an interactive narrator, maintaining narrative continuity, coaching skill use, and scaffolding problem-solving while retaining full clinical control of session goals.

Each quest corresponded to a specific therapeutic theme (e.g., self-regulation, organization, frustration tolerance) and was designed to be completed within a single 60-minute session. Quests typically consisted of three to four tasks. Early tasks introduced and practiced new concepts or skills, whereas later tasks in the quest required integration of all skills introduced within that session. A final, multi-session quest required the patient to flexibly combine skills acquired across the treatment course, reinforcing generalization and cumulative mastery.

Task completion was governed by explicit mechanics that linked therapeutic behaviors to in-game progress. To attempt a task, the patient was required to demonstrate a targeted skill or concept (e.g., identifying an emotion, breaking a task into steps, using a coping strategy). Successful demonstrations earned dice rolls, with thresholds set in advance and adjusted in real time by the therapist based on effort, engagement, and time constraints. Opportunities to earn additional dice or roll modifiers reinforced the use of previously learned skills, while unsuccessful attempts prompted brief review and repetition rather than punitive consequences. Mindfulness and other self-regulation strategies were explicitly embedded as alternative pathways to task completion following unsuccessful rolls.

OpenAI’s GPT-4o multimodal large language model was used as a preparatory tool to support the feasibility and consistency of the gamified intervention [15]. AI assistance was limited to generating draft quest outlines and narrative elements aligned with therapist-defined therapy themes and targeted skills. Clinical decision-making - including goal selection, skill sequencing, and adaptation - remained entirely therapist-directed. The model did not diagnose, assess, identify treatment targets, recommend therapeutic approaches, or determine the structure or duration of treatment.

Each week, the therapist used an ongoing AI prompt thread containing the established story arc and framework, manually entered the therapeutic theme and skills for that session, and requested narrative content (e.g., antagonists or symbolic objectives). All AI-generated material was reviewed, edited, and revised by the therapist before use, as outputs occasionally incorporated concepts inaccurately or inconsistently. Overall, AI functioned as a narrative support tool that reduced preparation burden while preserving therapist oversight and responsibility.

## Conclusions

In outpatient psychotherapy for youth with ADHD, core ADHD symptoms - such as distractibility, low frustration tolerance, and difficulty sustaining mental effort - can directly interfere with a patient’s capacity to participate in historical approaches to psychotherapy. When disengagement persists despite clinically appropriate content, clinicians should consider whether the structure and delivery of therapy itself require modification. A clinician-delivered, gamified adaptation of tabletop role-playing games is a practical and effective strategy for overcoming expected barriers to engagement. The narrative coherence, immediate and interval reinforcement, and increasing challenge may also enhance the overall effectiveness of therapy. Importantly, gamification does not eliminate the need for explicit skills coaching, guided insight, and therapeutic reflection. Patients may remain unable to independently identify, apply, or generalize skills without active clinician involvement. The clinical value of this gamified approach lies in its individualization and flexibility as a therapist’s tool, not as a standalone intervention: therapists can select and adjust therapeutic goals, concepts, skills, pacing, and difficulty in real time and across sessions to match evolving patient and family needs.

Finally, large language models may serve as useful adjuncts for generating creative narrative content or session materials, reducing preparation burden and supporting consistency. However, AI should not determine therapeutic targets, interpret clinical data, or guide treatment decisions. Clear boundaries, therapist review, and revision of AI-generated content are essential to preserve clinical judgment, developmental appropriateness, and therapeutic fidelity.
